# Notes and News

**Published:** 1887-08-27

**Authors:** 


### Notes and News.

Collected and Communicated.

On behalf of the fund for the proposed Cottage Hospital at Wellingborough, Dr. Howell Thomas has given a donation of £20, and Mr. G Plowman £10, by way of commencement. This is very well so far as it goes, but where are the local magnates—the brewers, lawyers, doctors, and shoe manufacturers? If Dr. Thomas gives £20, some of these should be prepared with four or five times that sum.

For the purpose of studying animal life under abnormal as well as normal conditions, an apparatus of iron and glass, in which a pressure of a thousand atmospheres can be developed, has been exhibited to biologists in France. By means of this apparatus deep-sea animals may be observed under their normal degree of compression.

It is intended to erect a Cottage Hospital in Falkirk, as a lasting memorial of the Queen's Jubilee. A committee has been appointed to carry out the object, consisting of Mr. Bolton, M.P., Mr. Sinclair, M.P., Provost Hodge and the magistrates of Falkirk, Councillors Cockburn and Baird, the clergy and doctors of the town, Colonel Nimmo, Mr. Mitchell of Millfield, Mr. Wilson of Bantaskine, and several others. The inhabitants of the town and neighbourhood are taking up the idea with much spirit, and it may be regarded as certain that the hospital will be an accomplished fact.

It is stated that the Queen having decided that the surplus of the Women's Jubilee Offering shall be devoted to the benefit of nurses or nursing establishments, has requested the committee to submit to her Majesty such proposals as may seem best calculated to carry out this intention.

Apropos of the above, Mrs. Bedingfeld, Lady Superintendent of the Belgravia Institute, writes : "I see in to-night's *Standard*, that her Majesty intends devoting the surplus of the Women's Jubilee Offering to the benefit of nurses and nursing establishments, and that she has appointed a committee to aid her in devising a plan for carrying out her wishes. Would it not be a good thing to bring the proposed National Pension Fund for Nurses before the Queen? She might easily vote a sum that would enable the Fund to be started at once. Several of my nurses will join if the rules of the Society meet with their approval ; and if the Queen would start it, it would be a grand thing with which to mark the Jubilee year."

The General Hospital at Launceston, Tasmania, is a Government institution, though to a large extent conducted on lines similar to those of the endowed and voluntary general hospitals of this country. The report for 1886, which has just reached us, furnishes interesting matter for comparison with similar reports issued by institutions at home. In one very important respect strong approval must be expressed regarding Colonial management. The average cost per bed per annum is the modest sum of £68 16s. $8\frac{4}{7}$d., an amount contrasting very favourably with that of some hospitals which might be mentioned in this hemisphere. When it is remembered that the hospital is hardly if at all assisted by voluntary contributions, but has the Fortunatus purse of Government to rely upon, the economy of management is the more surprising and gratifying. The total cost of the alcohol consumed in the hospital during the year was £81 11s. 10d., a diminution of £14 15s. 10d., as compared with the amount spent in the previous year. The hospital supplies a district containing a population of 54,000, and has an average of in-patients of about 70 daily, the total number of in-patients treated during the year being 925. The out-patients furnish a marked contrast to the same class in this country, numbering in the year not more than 512. The gross expenditure for 1886 was £5,162 10s. 4d., and of this no less than £1,241 11s. 10d. was contributed by patients' fees. It is interesting to note the classification of diseases adopted in the hospital, and to compare the relative severity of different diseases with that prevailing in our more variable climate. Diphtheria and typhoid furnish a large contingent, though the number of deaths under these headings is strikingly small. Ague and dysentery are rare, but rheumatism is decidedly frequent. Gout appears to be uncommon, but cancer by no means so. Diseases of the respiratory organs, notably phthisis, figure much more largely than might have been expected. It is worthy of note that diabetes is placed in the class of diseases of the urinary organs. The report is intelligent and exhaustive, and of much interest to all those concerned in the support and management of hospitals.

THE following vacancies are announced this week, the amount of the salary being placed in each case after the name of the institution :—

**Resident Medical Officers.**

Brighton and Hove Lying-in Institution. House Surgeon.—£120.
Bristol Royal Infirmary. Resident Medical Officer.—£80.
Denbighshire Infirmary. House Surgeon.—£85.
Female Lock Hospital, Harrow Road. Assistant House Surgeon.
Gainsborough Friendly Societies. Resident Officer.—£160 and Midwifery Fees.
General Hospital, Birmingham. House Governor.—£300.
Hulme Dispensary. House Surgeon.—£130.
Liverpool Dispensaries. Assistant Surgeons.—£80.
Manchester Royal Infirmary. Resident Surgical Officer.—£150.
Memorial Hospital, Jarrow. House Surgeon.—£130, £150, £175.
Portsmouth Hospital. £60.
St. Saviour's Union, Southwark. Assistant Medical Superintendent.—£130.

**Non-resident Medical Officers.**

Bristol Royal Infirmary. Obstetric Physician.
Cardiff Urban Sanitary District. Medical Officer of Health.
Eveline Hospital (Children). Out-patient Surgeon.
Teignmouth Urban Sanitary District. Medical Officer of Health.
Victoria Park Chest Hospital. Assistant Physician.

**Secretary, etc.**

Norfolk and Norwich Hospital.

**Matrons, etc.**

Royal Hospital for Incurables, Donnybrook, Dublin. Lady Superintendent.—£80.
Royal South Hants Infirmary. Lady, single.—£75.

**Trained Nurses.**

Bradford Infirmary. Assistant Nurse.—£18 with Uniform.
Burlington House, 11, Boundary Road, N.W. Several Trained Nurses wanted. Apply Lady Superintendent.
Cottishall Cottage Hospital, near Norwich. Trained Surgical Charge.—£20.
West London Hospital, Hammersmith Road. Fully-qualified.—£24.

---

IT is announced that the out-patients' department of Westminster Hospital will be closed for cleaning purposes for three weeks, from Monday, August 29th, to September 17th, inclusive.

---

LAST week, according to the Registrar-General's report, there were twenty-three deaths from scarlet fever in London, against twenty-five and twenty-one in the two previous weeks. The various metropolitan asylum hospitals contained 699 cases of scarlet fever at the end of the week. Up to going to Press this number has risen to 720. Scarlet fever is undoubtedly on the increase, though as yet there is nothing to justify alarm. The public will do well to keep doors and windows open, and to use disinfectants freely.

---

THE late Mr. Robert H. Arkley, of Dunninald, has bequeathed the munificent sum of £5,000 for the purpose of building and maintaining a convalescent home in connection with the Montrose Infirmary. It is hoped that this may form the nucleus of a fund for providing convalescent aid on a scale sufficient for the needs of the town and surrounding districts.

---

HER Majesty the Queen on Tuesday conferred the decoration of the Royal Red Cross upon the following nursing sisters :—Sister Louisa Jane Mackay, who served in South Africa and Egypt ; Sister A. B. Holland, who served in the hospitals at Netley, Chatham, and Portsmouth ; Sisters Edith King and Emma Durham, who were employed by the Stafford House Committee in the late Zulu war, and in various hospitals.

THE Directors of the Edinburgh Royal Hospital for Sick Children have unanimously resolved that one of the wards of the hospital shall be specially set apart for the treatment of surgical cases ; that this arrangement shall continue in force for three years from 1st November next, and at the expiration of that time shall be subject to reconsideration. Dr. Joseph Bell, F.R.C.S.E., has been appointed surgeon to the ward, Professor Annandale remaining consulting surgeon as at present. Dr. James Hutcheson and Dr. Ross Robertson have been elected to the office of resident physician for a period of six months.

---

LADY BROOKE laid the foundation stone of the Southend Hospital on Saturday afternoon, in the presence of a large and interested company. The hospital owes its inception to the medical men of the neighbourhood,—a proof, if any were wanted, that the members of the medical profession are not always thinking of their own pecuniary interests. So long ago as 1871, Mr. Hayward proposed that a small hospital should be erected ; but, like many other adventurous spirits, he was "before his age," and the suggestion evoked no public response. The Jubilee year has, however, brought the question to a crisis, and the result was the laying of the memorial stone on Saturday. The total cost of the building, with furniture, will be nearly £2,000, of which sum about £1,500 has been received or promised. The arrangements for the day were carried out by the general committee, of which Dr. W. R. Warwick is chairman. Among those present were Lord Brooke, the Rev. J. W. Herbert, vicar of the parish, and most of the principal inhabitants of the town and district, as well as many strangers. Lady Brooke placed a donation of £20 on the stone, which, with a collection made at the same time among the visitors, raised the amount contributed during the day to nearly £100.

---

THE Bournemouth Hospital scheme is still expanding. The original idea was to have a red-brick building with stone facings, and to spend £7,000 upon it. The line was to be drawn absolutely at £7,000. A beautiful town, however, demands beautiful public buildings, and such a degree of public spirit on the part of the inhabitants as will insist upon them. That spirit does not seem to be wanting in Bournemouth. A short time ago the £7,000 expanded to £9,000, with a correspondingly improved scheme. Now it is resolved that the brick building with stone facings is not worthy of the name and fame of Bournemouth, and so a solid structure of stone is to be raised, and the handsome sum of £10,000 is to be spent upon it. Doubtless some of the inhabitants who devote their leisure moments to the amusement of paring the public cheeses will have something to say on the subject. But the more generous spirits who have risen to the dignity of the occasion need not fear the general verdict when the scheme is completed. It would be discreditable in the highest degree to put up a mean public building of any kind in a town so universally admired as Bournemouth. Men, like other and lower organisms, can by no means resist the developmental force of their environment.

THE members and friends of The Hospitals Association are reminded that a reference library is being formed of books and pamphlets on all subjects connected with the construction, management, and maintenance of hospitals and other medical charities. Gifts of books or pamphlets will be thankfully received and duly acknowledged by Mr. Howard J. Collins, Secretary of The Hospitals Association, Norfolk House, Norfolk Street, Strand, W.C.

---

MESSRS. DOWN, St. Thomas's Street, have supplied some useful cases for the nurses at the Royal Berks Hospital. They are about the size of a lady's knitting case, and are fitted with enema syringe, in a waterproof bag, bone spatula, medicine and drop glasses, silver catheter, thermometer, dressing forceps, scissors, probe, box of safety-pins, hypodermic syringe, suture needles and silk, with room for tapes, needles, and thread, or other small requirements of a nurse.

---

A DEATH-RATE of 3·7 per cent. was reported by the secretary of the West Bromwich Hospital at the annual meeting on Tuesday. The Mayor, Alderman Farley, who presided, congratulated the hon. sec. on the exhaustive character of his report; and also upon the fact that there was a balance on the right side of £45 7s. 6d. at the end of the year. A cash balance, however small, is a pleasant consideration.

---

THE delegates of the Hospital Saturday Fund held a special meeting at the Board Room, 41, Fleet Street, on Saturday evening, Mr. James MacGregor presiding. The constitution of the Morley Convalescent Home for London Workmen, at St. Margaret's Bay near Dover, was under discussion. The Home was originally established by the Fund, and has recently been very considerably extended. After mature deliberation an amended constitution was unanimously agreed upon. The constitution as amended, of which a copy has been furnished to us, provides for the equitable and economical management of the institution. It is most satisfactory to find working men striving heartily and steadily to provide themselves with convalescent homes. For the payment of the modest sum of five shillings per week, workmen who have established claims upon this Home may not only enjoy its advantages, but may feel that those advantages are to all intents and purposes of their own providing. Self-help is the best of all help.

---

£30,000 is the substantial sum which the Harrogate "Bath Hospital and Convalescent Home," now in course of erection, is estimated to cost. Experts declare that the plans cannot be completed for the money—a very probable eventuality. Patients from England, Ireland, Scotland, and Wales were treated at the hospital last year; and with the vastly increased facilities afforded by the new buildings, the hospital will entirely lose any merely local character it may hitherto have possessed, and become altogether a national institution. Most readers know the objects for which patients are sent to Harrogate, viz., to drink the waters and take the baths. But a visit to Harrogate requires money, and however necessary the treatment may be, for members of the lower, middle, and poorer classes it is entirely unavailable without the "needful." The "Bath Hospital and Convalescent Home" are being built for the purpose of meeting the necessities of those who require the Bath and "Harrogate water" treatment all over the kingdom. The hospital proper will accommodate seventy-five patients, and the convalescent home fifty. They are both under one roof, and constitute one large and handsome building, which, with its tower of a hundred feet in height, will, when completed, be one of the finest public buildings in the West Riding. The people of Harrogate and the neighbourhood may well be praised for the generous and open-handed liberality which has inspired them to undertake so vast a scheme, but it will be nothing less than a national scandal if they are left to complete it alone. The hospital is for the gouty and rheumatic poor of the nation; and all those philanthropists who are interested in that large and unfortunate class should make a point of giving substantial aid to the building fund now, and of subscribing generously to the maintenance fund at the proper time.

---

AT Berlin, the scaffolding erected before a hospital which was being renovated has given way, and the architect and six workmen have been killed.

---

A RATHER important move, says the *Madras Times*, has been made by the medical profession in Calcutta, which will be watched with interest, and probably followed up, in other chief cities in India. Dr. Birch, at a meeting of the Calcutta Medical Society, brought forward a resolution, that, in the opinion of the Society, a Medical Registration Act for the town of Calcutta is urgently needed. Dr. Birch quoted several instances within his experience of injury done by unqualified practitioners. He said the question was, Is Calcutta ripe for the introduction of such an Act? In his opinion, it was not only ripe but over-ripe. Qualified medical relief is obtainable by everybody, either for nothing, medicines included, at any public hospital or charitable dispensary, or may be had for a few annas to the orthodox gold mohur, by those who desired it. By over-ripe, he referred to the legions of vain and ignorant pretenders, from the broken-down huckster to the aristocratic quack. The Medical Act of England, with certain modifications, he said, might be made applicable to Calcutta. The doctor would strictly limit the operations of the Act to the town of Calcutta, because in the Mofussil there is not the same plenitude of medical men, and they should not deprive a poor man of what in his ignorance he esteems a blessing. Dr. Macleod said it was well known that the municipality possessed the power of licensing medical practitioners. He was not aware if any steps were taken to ascertain whether the persons licensed were justified. Perhaps arrangements might be made by which this might be done, and this would fulfil all the purposes. He threw this out as a suggestion, and perhaps the New Municipal Law at present under consideration in the Bengal Council might add clauses to meet the object of the present proposal. The further discussion was postponed to the next meeting of the Society.

